# Aesthetic Restoration of an Old Composite Layering Using Digital Zirconia Veneers via an Indirect Technique: A Clinical Report

**DOI:** 10.7759/cureus.89000

**Published:** 2025-07-29

**Authors:** Zare G Donald, Oulghazi Ichraq, Amal El Yamani

**Affiliations:** 1 Prosthodontics, Faculty of Dental Medicine, Mohammed V University, Rabat, MAR

**Keywords:** cad cam, ceramic veneers, resin composite, zirconia etching, zirconia prosthesis

## Abstract

This article presents a clinical case where layered zirconia veneers were used to restore the patient's function and aesthetics. Composite direct layering restoration is a difficult therapeutic option that can lead to aesthetic and functional failure. Zirconia has come a long way since its introduction, and now layered zirconia veneers have become a popular option in prosthodontics due to their exceptional mechanical properties and aesthetic appeal. The described case outlines all the steps involved in a partial zirconia rehabilitation to achieve optimal results.

## Introduction

Clinical situations such as carious lesions, tooth discoloration, diastemas, crown fractures, and tooth misalignments can negatively impact dental aesthetics and the overall appearance of a smile. To address these concerns, two primary aesthetic treatment options are commonly used: direct resin-based composite restorations and indirect ceramic restorations.

Compared to indirect ceramic restorations, direct composite restorations offer several advantages, including single-visit treatment, reduced chair time, durability, and ease of repair. However, achieving long-term functional and aesthetic outcomes requires careful case selection, effective isolation, appropriate material choice, good optical and mechanical properties of the composite, operator experience, accurate shade matching, proper finishing and polishing, and regular follow-up visits. If these steps are not properly followed, complications such as fractures, chipping, marginal discoloration, or shade mismatch may occur [1].

Dental veneers are thin prosthetic coverings bonded to the enamel surface, designed to improve the color, alignment, and shape of the teeth. Ceramic veneers have become increasingly popular due to their minimally invasive nature, excellent biocompatibility, long-term durability, and superior aesthetic performance. Their clinical indications have expanded considerably, making them the treatment of choice for indirect anterior restorations. This shift reflects a modern trend toward conservative dentistry and minimally invasive treatment principles [2].

Ceramic veneers are particularly indicated for managing moderate intrinsic discolorations, such as those caused by aging, tetracycline staining, fluorosis, or enamel hypoplasia. They also offer effective solutions for restoring fractured, worn, or traumatized teeth, as well as for correcting minor anatomical anomalies and mild malocclusions [3].

Although no-prep veneers exist, most authors agree that minimal tooth preparation is necessary. This allows for the removal of aprismatic enamel, which is less favorable for bonding, and avoids overcontouring that could be detrimental to periodontal health. The literature describes four main types of tooth preparation: 1) window preparation, which preserves the incisal edge; 2) feather preparation, which involves a buccopalatal preparation without reducing the incisal length; 3) bevel preparation, which includes a slight incisal reduction (0.5-1 mm); and incisal overlap preparation, which involves greater reduction (approximately 2 mm), allowing the veneer to wrap around the palatal aspect of the tooth [4].

Zirconia has become a material of choice in recent years due to its non-metallic color, versatility, and exceptional fracture resistance, particularly in terms of flexural strength. It has demonstrated reliability in a wide range of clinical applications, whether used in monolithic restorations without cosmetic layering or in aesthetic cases involving pressed or layered ceramics on a zirconia framework [5,6].

The first generation of zirconia, known as 3Y-TZP, is characterized by a white color and high opacity. It is typically used as a prosthetic framework to be veneered with cosmetic ceramics. However, its major drawback is the risk of chipping, meaning fractures or cracks in the veneering ceramic due to occlusal forces [5-7].

Chipping remains a significant limitation of these restorations, prompting the development of new types of zirconia for monolithic use. The second generation of zirconia, still stabilized with 3% yttria, offers improved optical properties and greater translucency. Although it provides acceptable aesthetics for posterior use, it remains inferior to other ceramics in the anterior region. Additionally, this generation is vulnerable to low-temperature degradation, which accelerates aging. Therefore, to enhance aesthetics, this zirconia often requires cosmetic ceramic layering [7].

The third generation of zirconia is doped with 5 mol% yttria, resulting in partially stabilized zirconia with around 50% cubic phase. This phase is isotropic in all crystallographic directions, which reduces light scattering at grain boundaries and improves translucency. Unlike earlier generations, this type of zirconia remains stable at room temperature and is not susceptible to low-temperature degradation or transformation toughening. Although its mechanical properties are reduced compared to 3Y-TZP, its long-term structural stability is a clear advantage [8].

For zirconia bonding, two main strategies are used. The first is self-adhesive resin cements, which are applied to the internal surface of the restoration, followed by placement and a short flash of light curing (2 seconds) to ease removal of excess cement, then final polymerization under pressure. This method is efficient, but its bond strength is similar to that of resin-modified glass ionomer cements [9]. The second method is adhesive resin cements with 10-methacryloyloxydecyl dihydrogen phosphate (MDP), which requires sandblasting of the internal surface (50 µm particles at max 2 bar), rinsing, application of silane (air-dried), and bonding using an MDP-containing adhesive cement [9].

Proper sandblasting is essential to avoid introducing microcracks, phase transformation, or reduced fatigue resistance of the restoration [10].

Dental bonding must be performed under clean conditions, free from saliva and blood. If dentin sealing was performed during preparation, the surface must be either sandblasted with alumina and etched with phosphoric acid before drying, or treated according to the full protocol of the selected adhesive system (etch-and-rinse, self-etch, or universal adhesive), strictly following the manufacturer’s instructions [11].

## Case presentation

**Figure 1:**
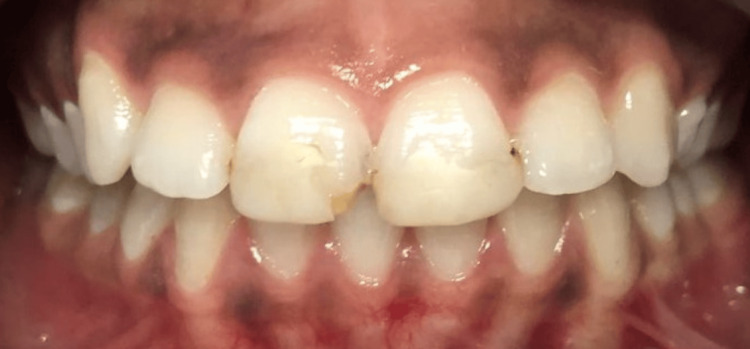
Initial intraoral clinical findings showing old and deteriorated composite restorations

The patient, a 22-year-old female in good general health, presented with central incisors 11 and 21, which had old, deteriorated composite restorations using direct monochromatic layering technique. The primary concern was the aesthetic restoration of these teeth (Figure 1).

Upon initial examination, there was no sign of decay, only unesthetic and defective composite restorations. The available treatment options included composite layering, full restoration with all-ceramic crowns, or ceramic veneers. Since there was no evidence of carious pathology and the patient preferred a durable restoration with better mechanical properties over composite layering, it was decided to restore the anterior teeth with veneers to improve the smile aesthetics while preserving as much healthy tooth structure as possible.

Unfortunately, the patient's medical coverage did not allow us to choose either high translucency zirconia or lithium disilicate. Therefore, we opted for second-generation zirconia layered with feldspathic ceramic.

Operative protocol

**Figure 2:**
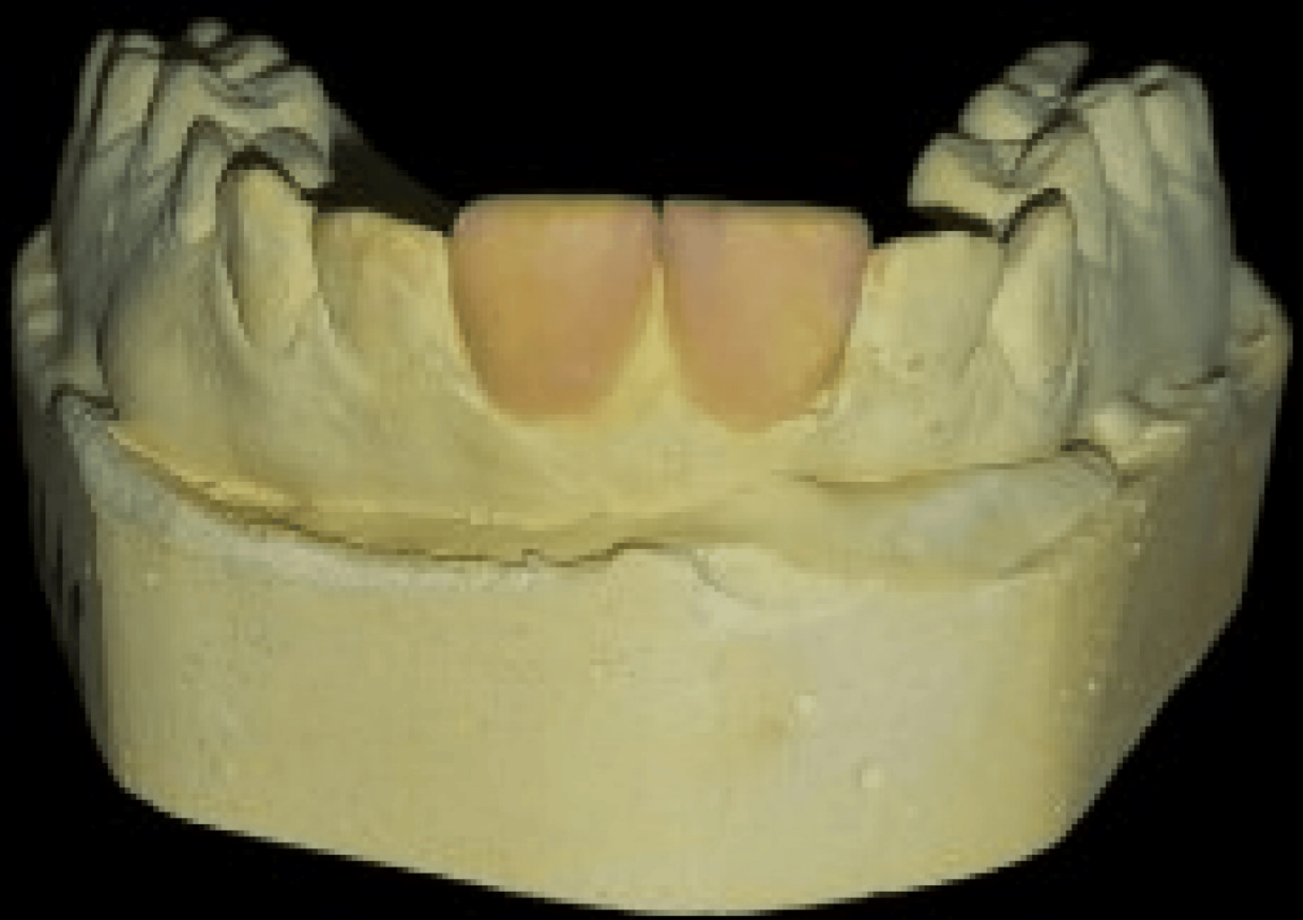
Diagnostic wax-up for aesthetic outcome simulation

A wax-up of teeth 11 and 21 was created in the laboratory using the primary impression taken with alginate (Figure 2).

**Figure 3:**
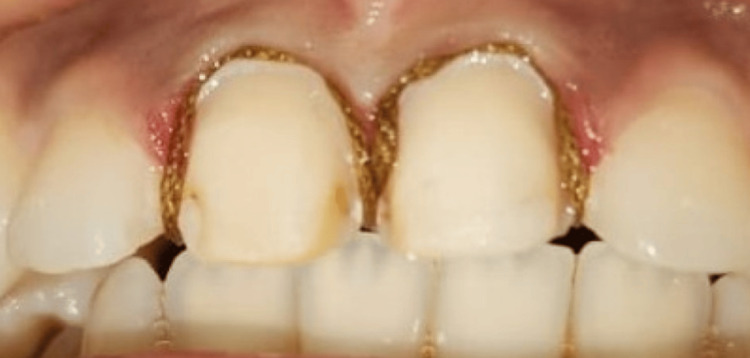
Dental preparation with palatal return for zirconia veneers

The preparation of the teeth involved palatal coverage. A 1 mm reduction of vestibular wall is minimal with a 1.5 - 2 mm reduction of incisal (Figure 3). Supragingival chamfer margins were prepared using a tapered round diamond round-end fissure burs. The contacts were not crossed

The palatal finish line of the incisal overlap should be positioned outside the occlusal impact points and avoided in areas of greatest palatal concavity to minimize stress on the ceramic.

**Figure 4:**
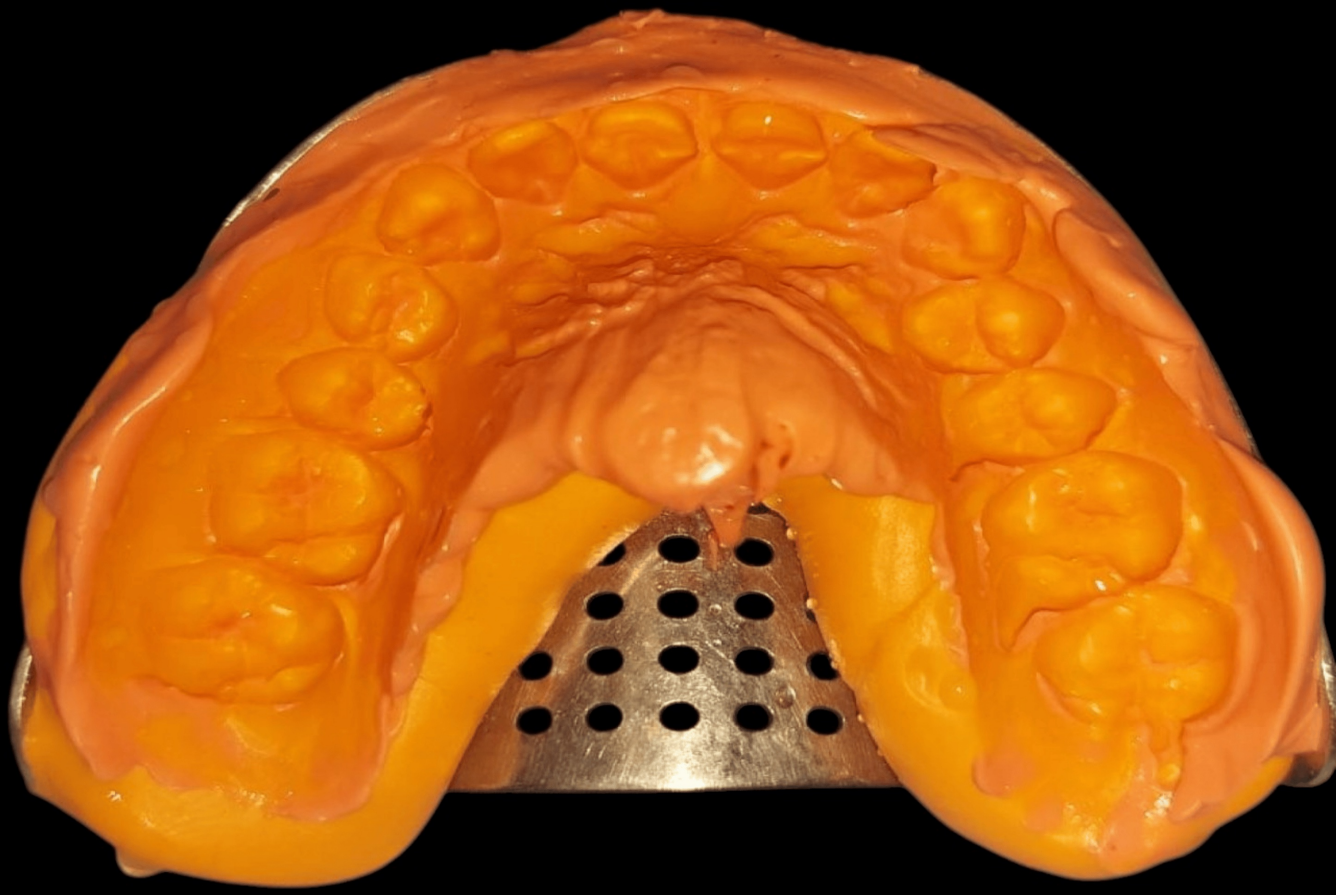
Secondary impression with addition silicone

A retraction cord was inserted in the gingival sulcus to provide an accurate thickness of the impression material. An impression was taken using additional silicone in a one-step putty wash technique for the prepared teeth to fabricate custom veneers (Figure 4).

Given the substantial preparation, self-polymerizing resin temporaries were placed and adjusted before releasing the patient. 

Computer-aided design (CAD) and fabrication of zirconia veneers

**Figure 5:**
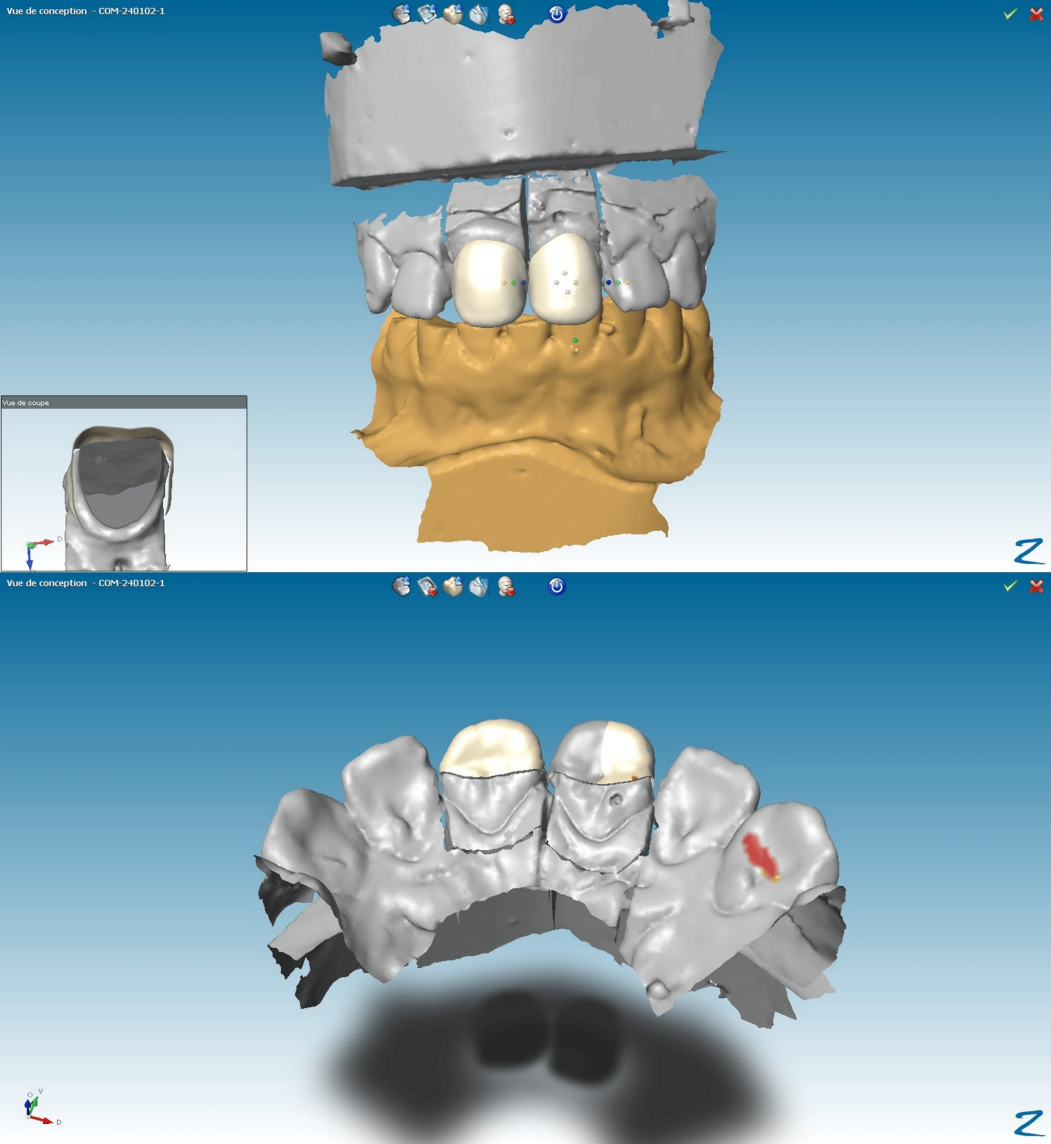
Digital scanning of the model followed by computer-aided design (CAD) of veneers with palatal coverage

The models obtained were scanned for the digital design of the zirconia framework. Once validated, this framework was manufactured using a three-axis subtractive computer numerical control (CNC) milling machine (Figure 5).

**Figure 6:**
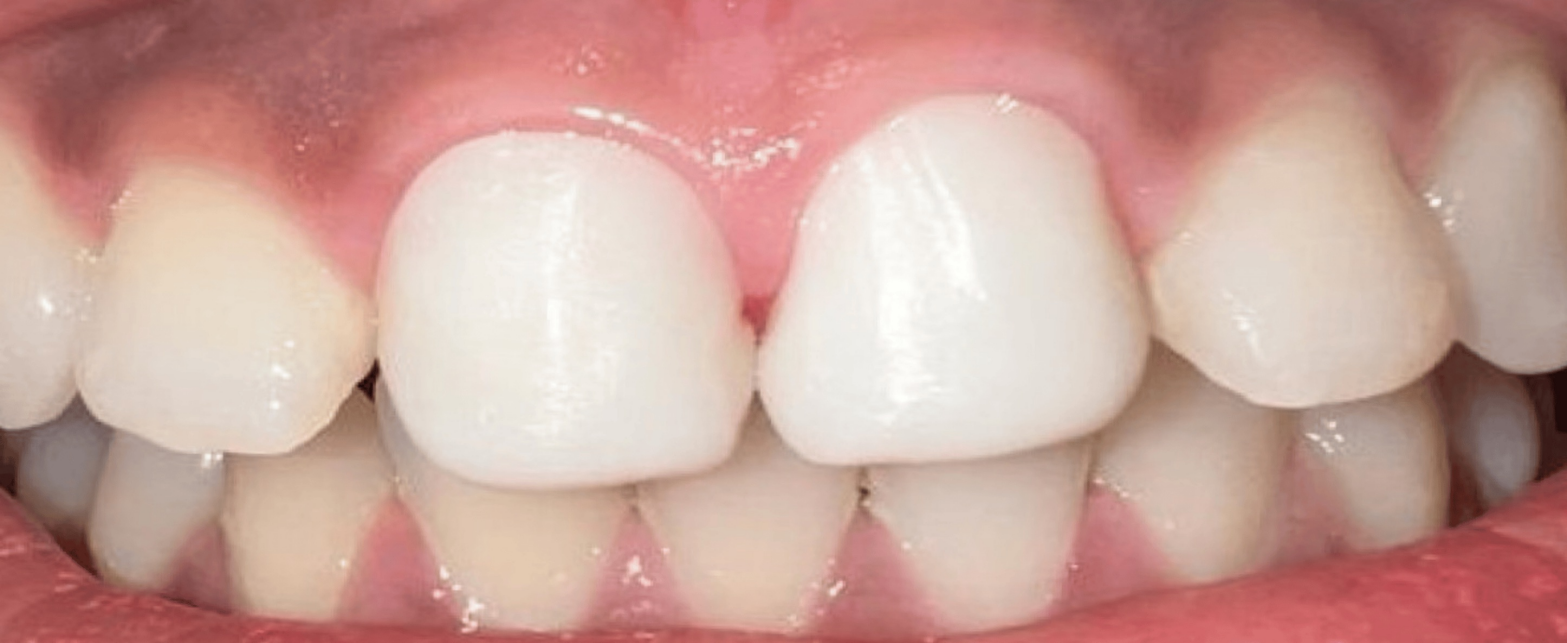
Zirconia framework fitting

The zirconia framework was tried in, excess contours were removed, and the shade was selected with visual assistance and photography. The patient was satisfied with the chosen shade (Figure 6).

**Figure 7:**
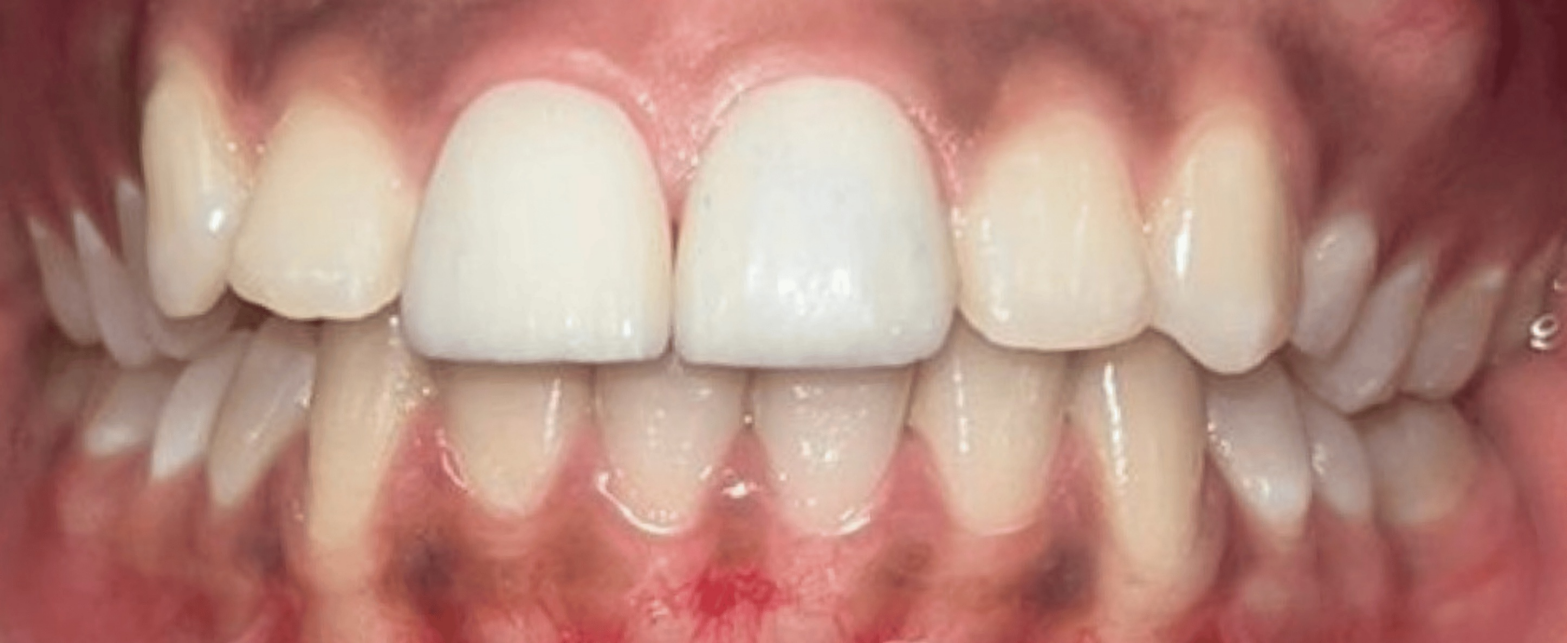
Fitting of veneers after cosmetic ceramic layering

The cosmetic ceramics were layered using the conventional hand-layering technique. Veneers were tested in the patient's mouth to ensure a perfect fit and proper occlusion (Figure 7).

**Figure 8:**
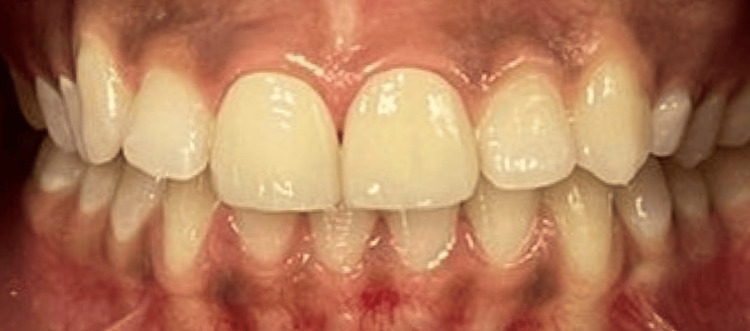
Final result with glazed zirconia

Once the patient was satisfied, a tribochemical treatment was performed in the laboratory. After placement of a rubber dam and gingival retraction cords, the veneers were permanently bonded using an adhesive bonding agent (Figure 8).

**Figure 9:**
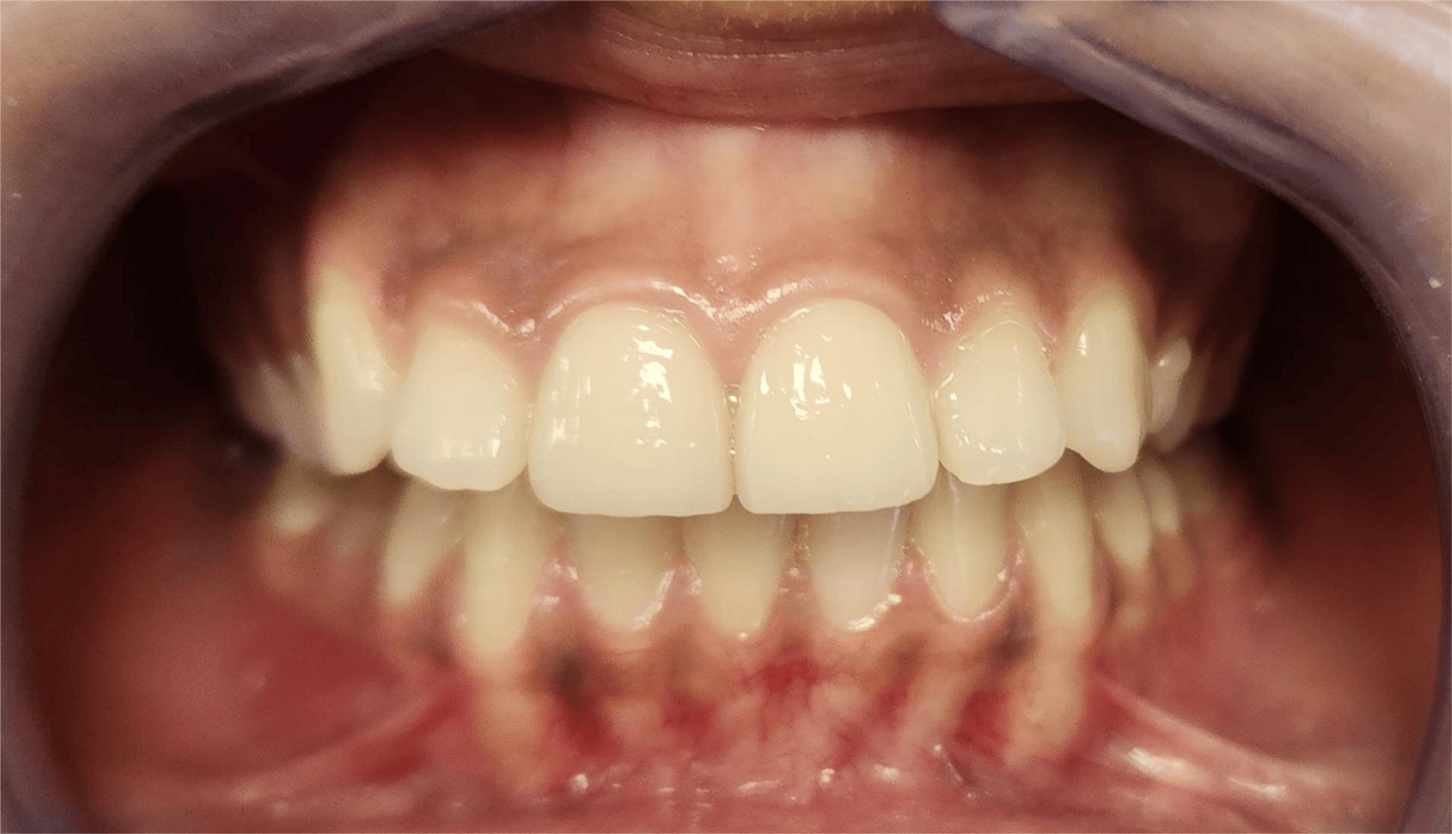
Inspection after 12 months follow-up

A follow-up examination after 12 months revealed that the patient had not experienced any discomfort. The aesthetic and functional integration of the prosthetic reconstruction was achieved due to the adherence and cooperation of the patient with the treatment plan (Figure 9).

## Discussion

In light of the increasing demand for aesthetic dental treatments and the evolution of restorative materials and techniques, the selection of a veneer type must balance aesthetic outcomes with the preservation of dental tissues. As described by Pini et al. [2], zirconia veneers may be indicated in cases of underlying tooth discoloration due to their superior masking ability compared to more translucent ceramics. Although less translucent than other ceramic options, zirconia frameworks veneered with cosmetic ceramics can achieve satisfactory aesthetic results, particularly when structural integrity and long-term performance are prioritized. In this case, the use of such veneers successfully addressed the aesthetic concern while preserving function and tissue, in accordance with modern conservative approaches [2].

However, high-translucency zirconia and lithium disilicate offer superior optical properties and clinical success rates compared to conventional veneered zirconia, and may be a more suitable option when aesthetics are paramount.

Composite layering allows for functional and aesthetic rehabilitation while preserving the tooth’s hard tissues. However, this technique significantly depends on the practitioner’s expertise in matching shade and morphology. Furthermore, poor composite material selection may lead to discoloration and long-term failure of the restoration [11].

Both ceramic and composite veneers restore aesthetics and tooth morphology but require the removal of some tooth structure. In cases of discolored underlying substrates, zirconia frameworks are often chosen for their ability to mask discoloration better than other ceramic options. However, zirconia veneers coated with cosmetic layering ceramics have demonstrated lower aesthetic properties in cases without discoloration or in pulpless abutments. In such scenarios, the use of a more translucent framework is preferable, as it allows light to diffuse more naturally through the tooth and surrounding tissues. In the present case, however, the aesthetic results were satisfactory for both the clinician and the patient. The veneers effectively corrected the aesthetic defect, and the mechanical properties of zirconia, combined with the preserved anterior teeth's proprioception, contributed to the overall durability of the restoration [12].

High-translucency zirconia and lithium disilicate are currently among the most aesthetically advanced ceramic materials for anterior restorations. Both provide greater translucency and optical integration than traditional veneered zirconia [13]. Despite these advantages, veneered zirconia remains a viable option, especially when mechanical strength is the primary concern. Clinical studies report success rates nearing 90% over 10 years for veneered zirconia crowns [14]. However, technical complications such as veneering ceramic chipping remain relatively common, with reported incidences ranging from 20% to 54%, depending on case complexity and prosthesis span [14]. The clinical success of layered zirconia restorations depends on multiple factors, including material properties, margin placement accuracy, appropriate veneering thickness, and precise CAD/CAM fabrication. Strong adhesive bonding between the zirconia core and the veneering ceramic is also crucial to prevent delamination or fracture [15]. When executed correctly, veneered zirconia restorations can deliver excellent aesthetic and functional outcomes, even in high-stress areas.

Framework architecture

In addition to zirconia’s intrinsic mechanical strength, the fracture resistance and long-term performance of veneered zirconia restorations depend heavily on optimized framework design. An anatomical framework with consistent ceramic thickness and rounded internal and interdental embrasures is essential to reduce stress concentrations and minimize chipping or fracture risk [16].

Digital fabrication accuracy

Studies suggest that zirconia restorations typically exhibit better marginal adaptation than internal fit, likely due to CAD/CAM limitations such as milling bur geometry and diameter. Nonetheless, both marginal and internal gaps usually fall within clinically acceptable ranges, with marginal gaps generally below 120 µm, in line with American Dental Association (ADA) guidelines [17].

Porcelain veneering technique

Zirconia-based restorations veneered with porcelain are prone to mechanical failures like chipping and delamination [18], which may occur as early as six months post-placement in some cases. However, in the current case, no chipping was observed after one year, indicating favorable performance under normal occlusal function.

The hand-layering technique was chosen for its ability to create a strong chemical bond between the veneering porcelain and zirconia core, which is vital for long-term durability. Among various veneering methods, feldspathic porcelain showed the most uniform and defect-free microstructure in cross-sectional analysis, outperforming pressable and CAD-on porcelains [19]. However, it is important to note that hand-layering has been associated with greater marginal discrepancies than press-over or CAD-on techniques, requiring a careful balance between aesthetic demands and mechanical integrity [16].

Finish line

Zirconia restorations prepared with chamfer finish lines tend to show larger marginal gaps than those with rounded shoulder margins, which promote better sealing and adaptation. In this case, a supragingival finish line was selected to facilitate soft tissue management and reduce the risk of gingival inflammation, particularly important in a patient with good oral hygiene and a favorable crown-root ratio [20].

## Conclusions

The aesthetic restoration of old composite layering using digital zirconia veneers via an indirect technique represents an advanced, evidence-based approach that balances durability, aesthetics, and minimal invasiveness. The success hinges on meticulous treatment planning, precise digital workflows, effective bonding protocols, and appropriate material selection. While zirconia veneers offer superior strength and biocompatibility, their aesthetic limitations necessitate veneering with aesthetic materials. The indirect technique enhances restoration quality by allowing optimal polymerization and finishing outside the oral environment. This approach is well supported by recent clinical reports and literature, demonstrating predictable, long-lasting, and highly aesthetic results.
